# Prognostic value of translocation 11;14 in patients with relapsed/refractory myeloma receiving anti-CD38 therapy

**DOI:** 10.1038/s41408-022-00769-4

**Published:** 2022-12-16

**Authors:** Ghulam Rehman Mohyuddin, Rajshekhar Chakraborty, Gregory S. Calip, Mustafa S. Ascha, Xiaoliang Wang, Samuel M. Rubinstein, Sascha Tuchman, Luciano Costa, Benjamin Haaland, Smith Giri, Hira Mian, Rafael Fonseca, Douglas Sborov

**Affiliations:** 1 Division of Hematology and Hematological Malignancies, Huntsman Cancer Institute, University of Utah, Salt Lake City, UT USA; 2 Department of Hematology, Columbia University, New York, NY USA; 3 Flatiron Health, New York, NY USA; 4 Department of Medicine, Division of Hematology, Lineberger Comprehensive Cancer Center, University of North Carolina at Chapel Hill, Chapel Hill, NC USA; 5 Division of Hematology and Oncology, University of Alabama at Birmingham, Birmingham, AL USA; 6 Department of Population Sciences, University of Utah, Salt Lake City, UT USA; 7 Department of Oncology, McMaster University, Hamilton, ON Canada; 8 Division of Hematology and Oncology, Mayo Clinic in Arizona, Phoenix, AZ USA

**Keywords:** Myeloma, Myeloma

Antibodies that target CD38 such as daratumumab and isatuximab have transformed the landscape of treatment for multiple myeloma (MM) [1]. The translocation t(11;14) is present in ~15% of patients newly diagnosed MM and is associated with a unique phenotype, including CD20 expression, lymphocytic morphology, scant cytoplasm, and low CD38 expression [2, 3]. Prior work has demonstrated that the prognosis for patients with t(11;14) may be slightly worse than standard risk disease when patients are treated with novel agents [4]. However, data on efficacy of anti-CD38 therapy for patients with and without t(11;14) are currently lacking.

To address this knowledge gap, we performed a retrospective cohort study using clinical and cytogenetic data from patients with relapsed/refractory MM in the Flatiron Health electronic health record (EHR)-derived database. We hypothesized that CD38 expression would be sufficient in those with t(11;14) to permit activity of anti-CD38 and that amongst patients receiving anti-CD38 therapies, outcomes would be similar in patients with t(11;14) when compared to those without the translocation.

We evaluated data from relapsed/refractory MM patients initiating treatment including daratumumab or isatuximab in their 2nd or later line of therapy between November 2015 and December 2021. We included patients ages 18 years and older with a clinically confirmed MM diagnosis between 2011 and 2021. Patients were also required to have documented cytogenetic testing by FISH with sufficient probes for high-risk cytogenetic abnormalities (HRCAs) and t(11;14) prior to or within 90 days following initiation of the index anti-CD38 line of therapy. The presence of t(11;14) and HRCAs defined as deletion 17p, amplification or gain 1q21, t(4;14), t(14;16), and t(14;20) was determined from FISH testing occurring at any point from the MM diagnosis through the index anti-CD38 line of therapy. Patients without t(11;14) and harboring no HRCA were defined as “wild-type”.

Our primary outcomes were progression-free survival (PFS) and overall survival (OS). Details of the definition and calculation of these outcomes are listed in the supplement.

Baseline demographic and clinical characteristics were compared between patients with and without t(11;14) using chi-square test and Wilcoxon rank-sum test for categorical and continuous variables respectively. Categories were combined or results were suppressed to avoid reporting any cell counts <5 for patient privacy.

We used the Kaplan-Meier method to calculate survivor functions for PFS and OS. All relevant tests were two-sided and p-values <0.05 were considered statistically significant. Analyses were conducted using R 3.6.1. A multivariate analysis was done adjusting for age, sex, functional status, ISS Stage, eGFR, autologous stem cell transplant receipt, lines of prior therapy, start year of therapy and type of CD-38 therapy used (example daratumumab/pomalidomide/dexamethasone, daratumumab/lenalidomide/dexamethasone, isatuximab based therapies, etc). Separate analysis was performed for patients with just t(11;14) and no other HRCA, as well as those without (t11;14) and no HRCA. A supplementary analysis was done to exclude patients that received venetoclax during follow-up.

An overall cohort of 1685 patients with MM initiating anti-CD38 therapy as 2nd or later treatment with a median follow-up of 22.6 months was identified. Patient characteristics are listed in Table 1.

**Table 1:** Descriptive characteristics of multiple myeloma patients initiating daratumumab- or isatuximab-based lines of therapy by t(11;14) status.

	All anti-CD38 therapy patients (*N* = 1685)		t(11;14)-negative (*n* = 1392)		t(11;14)-positive (*n* = 293)		*P*-value
	*N*	(%)	*n*	(%)	*n*	(%)	
**Age (years)**							0.879
Median (IQR)	67	(59–74)	67	(59–73)	67	(59–74)	
≤50	153	(9.1)	124	(8.9)	29	(9.9)	
51–65	600	(36.0)	501	(36.0)	99	(33.8)	
66–75	609	(36.1)	502	(36.1)	107	(36.5)	
76+	323	(19.2)	265	(19.0)	58	(19.8)	
**Sex**							0.037
Female	760	(45.1)	644	(46.3)	116	(39.6)	
Male	925	(54.9)	748	(53.7)	177	(60.4)	
**Race**							0.561
Non-Hispanic White	1042	(61.8)	861	(61.9)	181	(61.8)	
Non-Hispanic Black	257	(15.3)	206	(14.8)	51	(17.4)	
Non-Hispanic Asian	35	(2.1)	29	(2.1)	6	(2.0)	
Hispanic/Latinx	117	(6.9)	103	(7.4)	14	(4.8)	
Other race/ethnicity	126	(7.5)	102	(7.3)	24	(8.2)	
Not documented	108	(6.4)	91	(6.5)	17	(5.8)	
**Practice type**							0.705
Academic	221	(13.1)	183	(13.1)	38	(13.0)	
Community	1424	(84.5)	1174	(84.3)	250	(85.3)	
**ISS stage**							0.105
I	369	(21.9)	290	(20.8)	79	(27.0)	
II	382	(22.7)	315	(22.6)	67	(22.9)	
III	441	(26.2)	369	(26.5)	72	(24.6)	
Not documented	493	(29.3)	418	(30.0)	75	(25.6)	
**Baseline ECOG PS**							0.590
0	464	(27.5)	376	(27.0)	88	(30.0)	
1	709	(42.1)	591	(42.5)	118	(40.3)	
2+	272	(16.1)	222	(15.9)	50	(17.1)	
Unknown	240	(14.2)	203	(14.6)	37	(12.6)	
**Baseline eGFR**							0.213
≥40 ml/min	673	(39.9)	551	(39.6)	122	(41.6)	
<40 ml/min	161	(9.6)	141	(10.1)	20	(6.8)	
Not documented	851	(50.5)	700	(50.3)	151	(51.5)	
**M-protein type**							<0.001
IgA	376	(22.3)	318	(22.8)	58	(19.8)	
IgD	8	(0.5)	6	(0.4)	2	(0.7)	
IgE	1	(0.1)	1	(0.1)	0	(0.0)	
IgG	953	(56.6)	809	(58.1)	144	(49.1)	
IgM	9	(0.5)	4	(0.3)	5	(1.7)	
Multiple Ig	5	(0.3)	4	(0.3)	1	(0.3)	
Not documented	333	(19.8)	250	(18.0)	83	(28.3)	
**Light chain**							0.408
Kappa	1004	(59.6)	833	(59.8)	171	(58.4)	
Lambda	653	(38.8)	538	(38.6)	115	(39.2)	
Not documented	24	(1.4)	17	(1.2)	7	(2.4)	
**High-risk cytogenetics**							
Deletion 17p	253	(15.0)	208	(14.9)	45	(15.4)	0.856
Amplification/Gain 1q21	491	(29.1)	419	(30.1)	72	(24.6)	0.058
t(4;14)	151	(9.0)	133	(9.6)	18	(6.1)	0.063
t(14;16)	74	(4.4)	62	(4.5)	12	(4.1)	0.785
t(14;20)	15	(0.9)	13	(0.9)	2	(0.7)	>0.999
**Number of HRCAs**							0.068
0 HRCA	952	(56.5)	773	(55.5)	179	(61.1)	
1 HRCA	527	(31.3)	438	(31.5)	89	(30.4)	
2+ HRCA	206	(12.2)	181	(13.0)	25	(8.5)	
**Index line of therapy number**							0.061
2^nd^	657	(39.0)	527	(37.9)	130	(44.4)	
3^rd^	455	(27.0)	376	(27.0)	79	(27.0)	
4^th^ or later	573	(34.0)	489	(35.1)	84	(28.7)	
**Anti-CD38 therapy type**							0.924
Daratumumab/bortezomib/dexamethasone	347	(20.5)	292	(20.9)	55	(18.8)	
Daratumumab/lenalidomide /dexamethasone	283	(16.8)	233	(16.7)	50	(17.1)	
Daratumumab monotherapy	101	(6.0)	81	(5.8)	20	(6.8)	
Daratumumab/pomalidomide/dexamethasone	426	(25.3)	355	(25.5)	71	(24.2)	
Daratumumab/carfilzomib/dexamethasone	96	(5.7)	80	(5.7)	16	(5.5)	
Other daratumumab-based therapy	417	(24.7)	339	(24.4)	78	(26.6)	
Isatuximab-based therapy	15	(0.9)	12	(0.9)	3	(1.0)	
**Autologous stem cell transplantation**							0.014
Ever	155	(9.2)	117	(8.4)	38	(13.0)	
Never	1530	(90.8)	1275	(91.6)	255	(87.0)	

In this sample of patients that received FISH testing, 293 (17%) patients had t(11;14). A lower proportion of t(11;14) patients had one or more HRCAs compared to patients without t(11;14) (39% vs. 44%), driven primarily by differences in 1q amp/gain (25% amongst patients with t(11;14) compared to 30% amongst those without).

Kaplan–Meier survivor functions for PFS are shown in Fig. 1. In the overall study sample, patients with and without t(11;14) had a similar median PFS, 16.6 months and 15.0 months respectively (Panel A, *p* = 0.24). Median PFS was also similar when comparing patients with t(11;14) and no HRCAs (19.9 months) to wild-type patients (19.6 months) (Panel B, *p* = 0.59). Similar associations were observed for these comparisons in OS (Fig. S1). Median OS for patients with and without t(11;14) was 49.8 months and 31.9 months respectively (*p* = 0.07).

**Fig. 1:**
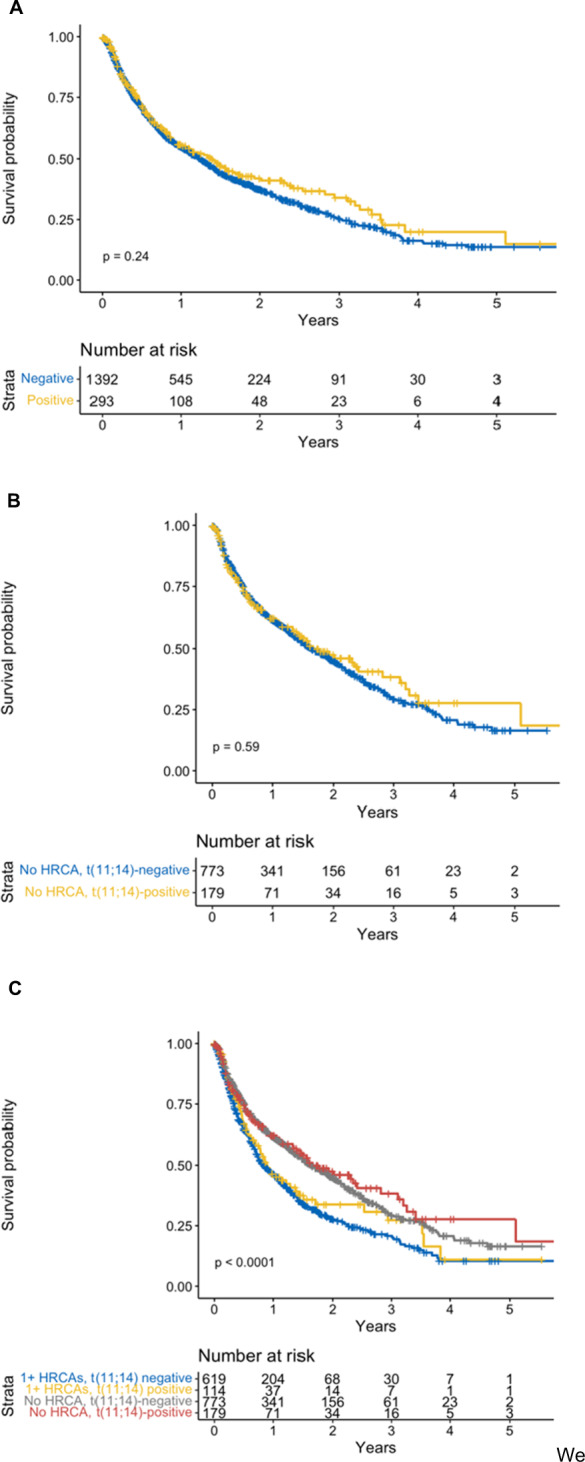
Kaplan–Meier describing survivor functions for progression free survival. **A** Stratified by t (11;14) negative or t(11;14) positive. **B** Stratified by t (11;14) negative with no other high risk cytogenetics and t (11;14) positive with no other high risk features. **C** Stratified into four groups based on the presence or absence of high risk cytogenetic abnormalities and the presence or absence of t (11;14).

In multivariable models comparing PFS in patients with t(11;14) to patients without t(11;14), we observed similar risks (HR 1.94, 95% CI 0.68-1.29, *p* = 0.70) after adjustment for confounders and presence of other HRCAs (Table S1). When comparing PFS in patients with t(11;14) and no HRCAs to wildtype patients, no statistically significant differences were observed (HR 1.05, 95% CI 0.66–1.66, *p* = 0.84).

In multivariable models comparing OS in patients with t(11;14) to patients without t(11;14), we observed similarly not significant risks (HR 0.71, 95% CI 0.47–1.08, *p* = 0.11) after adjustment for confounders and presence of other HRCAs (Table S1). When comparing OS in patients with t(11;14) and no HRCAs to wildtype patients, we also observed similar risks of overall mortality (HR 0.75, 95% CI 0.40–1.39, *p* = 0.36).

Patients with HRCA experienced inferior PFS if they did not have t(11;14) (HR 1.51, 95% CI 1.31–1.74, *p* < 0.01) but not if they had t(11;14) (HR 0.93, 95% CI 0.65–1.33, *p* = 0.70). On a multivariate model, patients with HRCA continued to experience inferior PFS if they did not have t(11;14) (HR 1.48, 95% CI 1.17–1.86, *P* < 0.01) but not if they had t(11;14)(HR 0.81, 95% CI 0.43–1.53, *p* = 0.52).

Patients with HRCA had a shorter overall survival if they did not have t(11;14) (HR 1.80 (95% CI 1.51–2.13, *p* < 0.01), but not if they had t(11;14) (HR 0.91, 95% CI 0.59–1.41, *p* = 0.67). On a multivariate model, patients with HRCA had an inferior overall survival if they did not have t(11;14) (HR 1.66, 95% CI 1.25–2.22, *p* < 0.01), but not if they had t(11;14) (HR 0.92, 95% CI 0.41–2.09, *p* = 0.85).

In a sensitivity analysis, we censored patients that later received venetoclax during follow-up and observed no changes in the direction or significance of the results (Supplementary Table 2).

In this large real-world study of outcomes of anti-CD38 therapy with relapsed/refractory MM, no significant difference in outcomes were observed between those who had t(11;14) compared to those who did not. As targeted therapies specific to patients with t(11;14) such as venetoclax are increasingly utilized in this patient population, it is important to recognize that anti-CD38 therapy should remain an important part of the treatment landscape for these patients, and that synergistic strategies incorporating bcl-2 inhibition and anti-CD38 targeting should be explored further in clinical trials.

Although previous ex-vivo studies have suggested that CD38 expression is decreased in patients with MM that harbors t(11;14) [3], we hypothesize that the CD38 expression is sufficient enough to allow the activity of anti-CD38 therapy in this setting. We also observed that in patients receiving anti-CD38 therapies, the presence of t(11;14) conferred a protective effect in those with HRCA. These findings were observed even after excluding patients with Gain/Amp 1q, which may commonly co-exist with t(11;14) [5]. The clinical and biological significance of this finding is unknown, and this requires further validation. In previous work using Flatiron, the presence of t (11;14) co-existing with 17p deletion in newly diagnosed MM was not a protective factor [4]. Furthermore, data from the Mayo Clinic has also indicated that patients with HRCA and t(11;14) do not have different outcomes compared to those with HRCA without t(11;14) [6]. It should be noted that in both these studies were studies in the newly diagnosed setting and rates of anti-CD38 usage were low. Thus, a unique effect of anti-CD38 therapy in abrogating poor outcomes of HRCA in patients with t(11;14) cannot be ruled out. It could also be that this finding simply reflects the higher proportion of patients with 2 or more HRCA compared in the non t(11;14) cohort compared to the t(11;14) cohort (13% vs 8.5%), rather than a true biological effect. As previous studies have evaluated the impact of t(11;14) for newly diagnosed MM rather than relapsed/refractory MM, it is unclear whether our findings represent anti-CD38 therapy abrogating a potential negative prognostic factor of t(11;14) in relapsed/refractory MM, or whether t(11;14) is fundamentally a “neutral” prognostic factor in patients with relapsed/refractory MM.

Limitations of our dataset include that it is limited to patients in the relapsed/refractory setting, as anti CD38 therapy has only recently begun to be incorporated in the newly diagnosed setting. Furthermore, our analysis on specific cytogenetic subsets of patients, such as those with HRCA but no Amp/Gain1q is limited by small numbers and should only be considered as hypothesis-generating. We also recognize that there were large numerical differences in overall survival between different subsets of patients, which did not approach statistical significance, but may still be of relevance. Furthermore, although the hypothesis of our study was dependent on the observation that CD38 expression may be lower in those with t(11;14), it has been observed that patients may continue to respond to daratumumab even when the CD38 expression by myeloma cells is low, perhaps owing to an immunomodulatory effect of these drugs [7]. It must also be noted that in order to isolate the effect of CD38 therapy on patients with t(11;14), our analysis would have benefited from a “control” group of patients with t(11;14) that did not receive anti-CD38 therapy, as we cannot isolate the incremental value that anti-CD38 therapy adds in patients with t(11;14) in our current analysis.

In summary, we demonstrate that the presence of t(11;14) does not appear to be an adverse prognostic factor amongst patients with relapsed/refractory MM receiving anti-CD38 therapy, with similar outcomes observed between those with or without t(11;14). Further prospective trials are needed to help delineate the magnitude of benefit that anti-CD38 therapy provides for patients with MM harboring t(11;14).

## Supplementary information

Supplemental Material

## Data Availability

Data may be made available with a reasonable request to the corresponding author.
