# Supplementary material for: Prognostic value of translocation 11;14 in patients with relapsed/refractory myeloma receiving anti-CD38 therapy
Source: Blood Cancer J. 2022 Dec 16;12(12):168. doi: 10.1038/s41408-022-00769-4 (PMC9758218; doi:10.1038/s41408-022-00769-4)
Supplement: Supplementary file 1 — Supplemental Material [file 41408_2022_769_MOESM1_ESM.docx]

| **Supplement Table 1.** Results from time-dependent Cox proportional hazards models for associations between t(11;14) and progression-free survival and overall survival | | | | | | |
| --- | --- | --- | --- | --- | --- | --- |
|  | **Crude, unadjusted model** | | | **Multivariable-adjusted model^a^** | | |
|  | **HR** | **(95% CI)** | **P** | **HR** | **(95% CI)** | **P** |
| **Progression-free survival** |  |  |  |  |  |  |
| *Reference: no t(11;14)^b^* |  |  |  |  |  |  |
| t(11;14) negative | 1.00 | Reference |  | 1.00 | Reference |  |
| t(11;14) positive | 0.90 | (0.75-1.07) | 0.242 | 0.94 | (0.68-1.29) | 0.704 |
|  |  |  |  |  |  |  |
| *Reference: wildtype* |  |  |  |  |  |  |
| No t(11;14), no HRCAs | 1.00 | Reference |  | 1.00 | Reference |  |
| 1+ HRCAs, t(11;14) negative | 1.51 | (1.31-1.74) | <0.001 | 1.48 | (1.17-1.86) | 0.001 |
| 1+ HRCAs, t(11;14) positive | 0.93 | (0.65-1.33) | 0.698 | 0.81 | (0.43-1.53) | 0.520 |
| t(11;14)-positive, no HRCAs | 0.94 | (0.74-1.19) | 0.605 | 1.05 | (0.66-1.66) | 0.844 |
|  |  |  |  |  |  |  |
| **Overall survival** |  |  |  |  |  |  |
| *Reference: no t(11;14)^b^* |  |  |  |  |  |  |
| t(11;14) negative | 1.00 | Reference |  | 1.00 | Reference |  |
| t(11;14) positive | 0.82 | (0.66-1.02) | 0.073 | 0.71 | (0.47-1.08) | 0.1116 |
|  |  |  |  |  |  |  |
| *Reference: wildtype* |  |  |  |  |  |  |
| No t(11;14), no HRCAs | 1.00 | Reference |  | 1.00 | Reference |  |
| 1+ HRCAs, t(11;14) negative | 1.80 | (1.51-2.13) | <0.001 | 1.66 | (1.25-2.22) | 0.001 |
| 1+ HRCAs, t(11;14) positive | 0.91 | (0.59-1.41) | 0.672 | 0.92 | (0.41-2.09) | 0.851 |
| t(11;14)-positive, no HRCAs | 0.87 | (0.64-1.18) | 0.368 | 0.75 | (0.40-1.39) | 0.358 |
|  |  |  |  |  |  |  |
| 1. Multivariable model adjusted for: age (continuous); sex (female, male); ECOG PS (0, 1, 2+, unknown); ISS stage (I, II, III, unknown); eGFR (≥40, <40, unknown), autologous stem cell transplantation (ever/never, time-varying), first anti-CD38 containing line number, year of first anti-CD38 therapy initiation, and anti-CD38 line of therapy type 2. Adjusted for presence of HRCAs: deletion 17p; amplification 1q21; t(4;14); t(14;16); t(14;20) | | | | | | |

| **Supplement Table 2.** Results from time-dependent Cox proportional hazards models for associations between t(11;14) and progression-free survival and overall survival, sensitivity analysis excluding patients that receive venetoclax within any line of therapy (n=51) | | | | | | |
| --- | --- | --- | --- | --- | --- | --- |
|  | **Crude, unadjusted model** | | | **Multivariable-adjusted model^a^** | | |
|  | **HR** | **(95% CI)** | **P** | **HR** | **(95% CI)** | **P** |
| **Progression-free survival** |  |  |  |  |  |  |
| *Reference: no t(11;14)^b^* |  |  |  |  |  |  |
| t(11;14) negative | 1.00 | Reference |  | 1.00 | Reference |  |
| t(11;14) positive | 0.88 | (0.73-1.07) | 0.191 | 0.94 | (0.65-1.36) | 0.740 |
|  |  |  |  |  |  |  |
| *Reference: wildtype* |  |  |  |  |  |  |
| No t(11;14), no HRCAs | 1.00 | Reference |  | 1.00 | Reference |  |
| 1+ HRCAs, t(11;14) negative | 1.55 | (1.34-1.78) | <0.001 | 1.57 | (1.24-1.99) | <0.001 |
| 1+ HRCAs, t(11;14) positive | 0.91 | (0.62-1.34) | 0.635 | 0.61 | (0.29-1.27) | 0.189 |
| t(11;14)-positive, no HRCAs | 0.93 | (0.72-1.20) | 0569 | 1.20 | (0.70-2.05) | 0.506 |
|  |  |  |  |  |  |  |
| **Overall survival** |  |  |  |  |  |  |
| *Reference: no t(11;14)^b^* |  |  |  |  |  |  |
| t(11;14) negative | 1.00 | Reference |  | 1.00 | Reference |  |
| t(11;14) positive | 0.85 | (0.68-1.08) | 0.180 | 0.78 | (0.49-1.24) | 0.286 |
|  |  |  |  |  |  |  |
| *Reference: wildtype* |  |  |  |  |  |  |
| No t(11;14), no HRCAs | 1.00 | Reference |  | 1.00 | Reference |  |
| 1+ HRCAs, t(11;14) negative | 1.84 | (1.55-2.18) | <0.001 | 1.79 | (1.33-2.41) | <0.001 |
| 1+ HRCAs, t(11;14) positive | 0.85 | (0.53-1.35) | 0.487 | 0.64 | (0.25-1.60) | 0.338 |
| t(11;14)-positive, no HRCAs | 0.93 | (0.68-1.29) | 0.680 | 0.98 | (0.50-1.60) | 0.338 |
|  |  |  |  |  |  |  |
| 1. Multivariable model adjusted for: age (continuous); sex (female, male); ECOG PS (0, 1, 2+, unknown); ISS stage (I, II, III, unknown); eGFR (≥40, <40, unknown), autologous stem cell transplantation (ever/never, time-varying), first anti-CD38 containing line number, year of first anti-CD38 therapy initiation, and anti-CD38 line of therapy type 2. Adjusted for presence of HRCAs: deletion 17p; amplification 1q21; t(4;14); t(14;16); t(14;20) | | | | | | |

**Figure S1** Kaplan-Meier survivor functions for overall survival [log-rank test]

**Panel A.** Stratification by (i) t(11;14) negative; (ii) t(11;14) positive

**
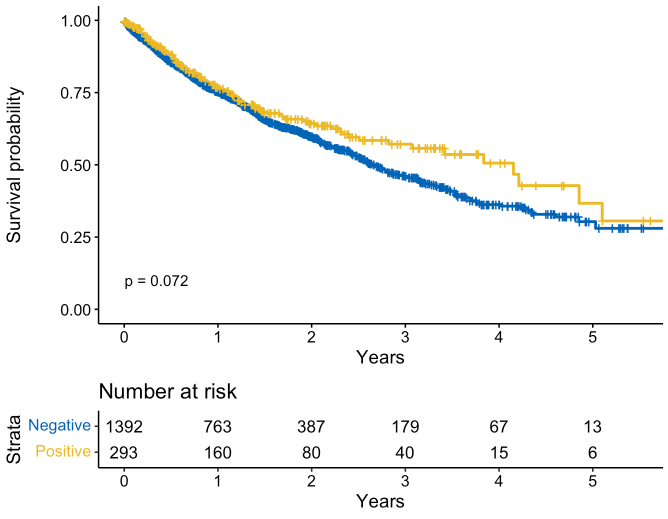
**

**Panel B.** Stratification by (i) No HRCAs, t(11;14) negative; (ii) t(11;14) positive, no HRCAs


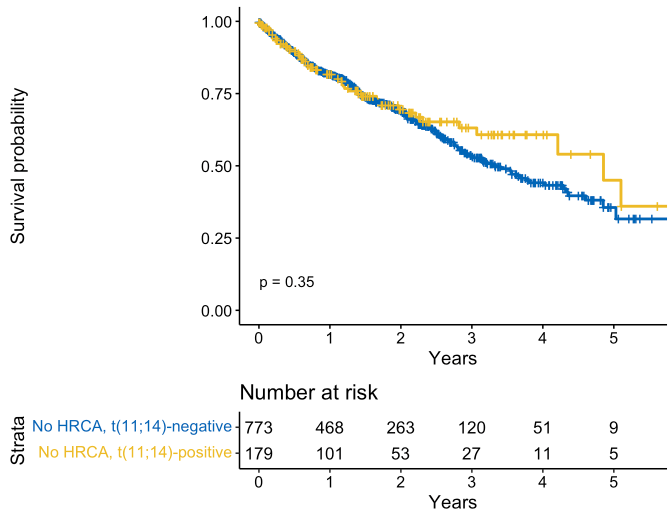


**Panel C.** Stratification by (i) No HRCAs, t(11;14) negative; (ii) 1+ HRCAs, t(11;14) negative; (iii) 1+ HRCAs; t(11;14)-positive; (iv) t(11;14) positive, no HRCAs


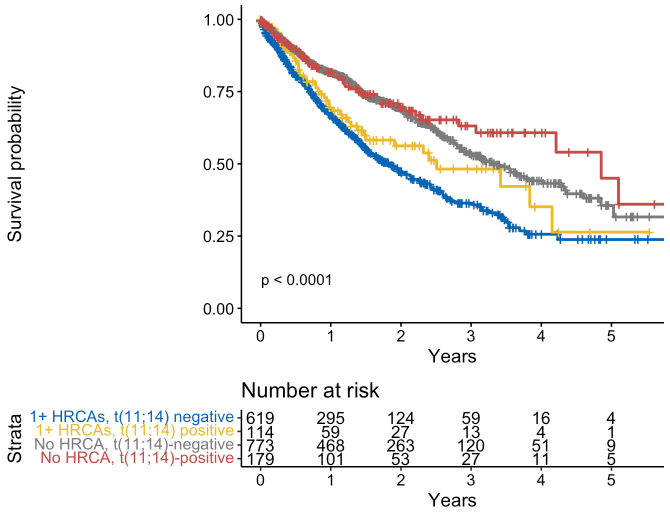


SUPPLEMENTAL INFORMATION:

Details of Progression Assessment:

Disease progression status is derived from laboratory measures of myeloma disease burden. First, each patient at treatment initiation is assigned a biomarker for tracking disease progression:

- First choice: serum M-protein measure. Must have measurable disease at baseline (serum M-protein ≥1 g/dL) to use serum M-protein for tracking progression.
- Second choice: urine M-protein measure. Must have measurable disease at baseline (urine M-protein ≥200 mg) to use urine M-protein for tracking progression.
- Third choice: free light chain (FLC) ratio.

Once a baseline biomarker for tracking disease progression has been defined, the patient is tracked longitudinally for a clinically meaningful increase in serum M-protein, urine M-protein, or the FLC ratio, based on International Myeloma Working Group (IMWG) criteria.^18,19^ However, it should be noted that the FH-derived progression algorithm does not consider the following IMWG criteria in ascertaining progression events:

- ≥ 25% increase in bone marrow plasma cell percentage from nadir and absolute increase ≥10%.
- Definite development of new bone lesions or soft tissue plasmacytomas or definite increase in the size of existing bone lesions or soft tissue plasmacytomas.
- Development of hypercalcemia that can be attributed solely to the plasma cell proliferative disorder.

In addition, only one of either serum M-protein or urine M-protein is used, while IMWG criteria would stipulate use of both serum and urine M-protein laboratory measures
